# Pyrroloquinoline quinone protects against murine hepatitis virus strain 3-induced fulminant hepatitis by inhibiting the Keap1/Nrf2 signaling

**DOI:** 10.1007/s10616-024-00627-0

**Published:** 2024-04-17

**Authors:** Zunguo Pu, Fei Ge, Yaqing Zhou, Aiming Liu, Chao Yang

**Affiliations:** 1 Department of Critical Care Medicine, Affiliated Haian People’s Hospital of Nantong University, Nantong, 226600 Jiangsu China; 2 Department of Gastroenterology, Haian Hospital of Traditional Chinese Medicine Affiliated to Nanjing University of Chinese Medicine, Nantong, 226600 Jiangsu China; 3 Key Laboratory of Liver Transplantation, Hepatobiliary Center, The First Affiliated Hospital of Nanjing Medical University, Chinese Academy of Medical Sciences, Nanjing, 210029 Jiangsu China

**Keywords:** Fulminant hepatitis, Pyrroloquinoline quinone, Keap1/Nrf2 signaling pathway, Oxidative stress, Acute liver injury

## Abstract

Fulminant hepatitis (FH) is a life-threatening clinical liver syndrome characterized by substantial hepatocyte necrosis and severe liver damage. FH is typically associated with severe oxidative stress, inflammation, and mitochondrial dysfunction. Pyrroloquinoline quinone (PQQ), a naturally occurring redox cofactor, functions as an essential nutrient and antioxidant and reportedly inhibits oxidative stress and exerts potent anti-inflammatory effects. In the present study, we aimed to evaluate the therapeutic efficacy of PQQ in murine hepatitis virus strain 3 (MHV-3)-induced FH and examined the underlying mechanism. An MHV-3-induced FH mouse model was established for in vivo examination*.* Liver sinusoidal endothelial cells (LSECs) were used for in vitro experiments. Herein, we observed that PQQ supplementation significantly attenuated MHV-3-induced hepatic injury by suppressing inflammatory responses and reducing oxidative stress. Mechanistically, PQQ supplementation ameliorated MHV-3-induced hepatic damage by down-regulating the Keap1/Nrf2 signaling pathway in vivo and in vitro. Furthermore, Nrf2 small interfering RNA targeting LSECs abrogated the PQQ-mediated protective effects against MHV-3-related liver injury. Our results deepen our understanding of the hepatoprotective function of PQQ against MHV-3-induced liver injury and provide evidence that alleviating oxidative stress might afford a novel therapeutic strategy for treating FH.

## Introduction

Fulminant hepatitis (FH, also called acute liver injury), a severe life-threatening clinical liver syndrome, is characterized by marked hepatocyte necrosis and severe liver damage. Currently, there is no effective treatment for FH (Staff 2016). The mortality rate of FH remains considerably high, although liver transplantation has been effectively employed to treat this disease (Rosen and Martin 2000). Therefore, it is critical to explore the pathogenesis of FH and identify effective treatment strategies. The mouse FH model induced by murine hepatitis virus strain-3 (MHV-3) has been widely employed in FH investigations, given the presence of sinusoidal thrombosis, hepatocellular necrosis, and elevated levels of fibrinogen-like protein 2 (Pope et al. 1995; Marsden et al. 2003; Levy et al. 1981).

It is well known that the liver exhibits an extraordinary ability to detoxify and eliminate diverse xenobiotics, including drugs and toxins, that can cause liver damage (Larrey 2009). Under pathological conditions, the liver is subjected to elevated levels of oxidative stress, resulting in the destruction of biological macromolecules, including DNA, proteins, and lipids (Chen et al. 2018; Tan et al. 2018). Both chronic and acute liver diseases are typically accompanied by an increase in reactive oxygen species (ROS) and an imbalance of oxidants and antioxidants (Cichoz-Lach and Michalak 2014; Jin et al. 2019), which play an important role in the liver fibrogenic response and the development of ischemia/regeneration. Therefore, exploring the mechanism underlying oxidative stress damage is pivotal for developing effective treatment strategies.

Pyrroloquinoline quinone (PQQ) is a powerful water-soluble antioxidant initially considered a natural bacterial cofactor (Rucker et al. 2009). PQQ reportedly plays an essential role in multiple physiological functions by ameliorating oxidative stress and senescence, including preventing estrogen deficiency-induced bone loss (Geng et al. 2019) and anterior cruciate ligament transection-induced knee osteoarthritis (Qin et al. 2019), as well as attenuating cachexia-induced muscle atrophy (Xu et al. 2018) and D-galactose-induced cognitive impairments (Zhou et al. 2018). However, whether PQQ supplementation can prevent FH development by inhibiting oxidative stress remains unknown.

Nrf2 (Nuclear factor-E2-related factor 2) has been widely detected in multiple organs in the body and plays an important role in defending against redox imbalance by regulating the expression levels of antioxidant genes (Yang et al. 2018; Nioi and Hayes 2004). Animal experiments have revealed that the mice exhibit notable aging phenotypes characterized by a decline in antioxidative pathways following knockout of Nrf2 (Hayes and Dinkova-Kostova 2014; Cheung et al. 2014). Therefore, we investigated whether PQQ supplementation could prevent the development of FH by inhibiting oxidative stress and DNA damage by upregulating the expression of Nrf2.

In the present study, we investigated whether PQQ could attenuate MHV-3-induced hepatic injury by suppressing oxidative stress. We demonstrated that PQQ exerted protective effects against MHV-3-mediated hepatocellular damage by inhibiting oxidative stress. Mechanistically, PQQ may alleviate MHV-3-induced acute liver injury by suppressing the Keap1/Nrf2 signaling pathway. In conclusion, our findings revealed, for the first time, the molecular mechanisms underlying the PQQ-mediated hepatoprotective effects in an MHV-3-induced FH model.

## Materials and methods

### Animals

Six-week-old male C57BL/6 mice were purchased from Charles River (Beijing Vital River Laboratory Animal Technology Co. Ltd., China). All mice were randomly divided into five animals per cage and fed a normal diet and a PQQ-supplemented diet (4 mg PQQ/kg in normal diet) (Huang et al. 2018) for 4 weeks at Nanjing Medical University Experimental Animal Base. PQQ disodium salt (referred to as PQQ, with a purity of 99%) was obtained from ECA Healthcare Inc. (Shanghai, China). All experimental procedures followed the ARRIVE guidelines (Kilkenny et al. 2010), were approved by the Committee on the Ethics of Animal Experiments (IACUC2001020), and were in accordance with the guidelines of laboratory mouse research at Nanjing Medical University.

### Virus and infection

MHV-3 was expanded to 1 × 10^7^ plaque-forming units (PFUs)/ml in murine L2 cells and stored at -80 °C until use. Mice received 10 PFU of MHV-3/mouse via intraperitoneal injection and subsequently sacrificed to obtain serum samples and harvest livers after 48 h. Serum levels of alanine aminotransferase (ALT) and aspartate aminotransferase (AST) were measured at Nanjing Medical University Medical Laboratory Animal Center.

### Biochemical measurements

Serum levels of total superoxide dismutase (T-SOD) (#A001-1; Nanjing Jiancheng Bioengineering Institute, Nanjing, China), reduced glutathione (GSH) assay kit (#A006-2-1; Nanjing Jiancheng Bioengineering Institute, Nanjing, China), and malonaldehyde (MDA) (#A003-1; Nanjing Jiancheng Bioengineering Institute) were measured according to the manufacturer’s instructions.

### Enzyme-linked immunosorbent assay

Serum levels of tumor necrosis factor (TNF)-α, fibrinogen-like protein 2 (FGL2), and interleukin (IL)-1β were determined using the tumor necrosis factor-α assay kit (#H052; Nanjing Jiancheng Bioengineering Institute, Nanjing, China), mouse FGL2 ELISA Kit (#E90512Mu; Uscn Life Science Inc., Wuhan, China), and interleukin-1β Assay kit (#H002; Nanjing Jiancheng Bioengineering TNFInstitute, Nanjing, China), respectively, according to the manufacturer's instructions.

### ROS level

After sacrificing mice, liver tissue samples were isolated and ground to obtain 10% tissue homogenate to detect the active oxygen (ROS) using the DCFDA/H2DCFDA-Cellular ROS Assay Kit (#ab113851; Abcam) according to the manufacturer’s instructions.

### Liver sinusoidal endothelial cell (LSEC) isolation

LSECs were isolated as previously described (Sun et al. 2017) and exposed to a gradient series of PQQ (0, 0, 0.1, and 0.2 μM) for 6 h. Considering groups treated with the last three PQQ concentrations, LSECs were treated with 1,000 PFUs of MHV-3 for 4 h and proteins and RNA were collected for analysis. LSECs were divided into four groups and processed as follows. Group 1 was transfected with NC small interfering RNA (siRNA). Group 2 was transfected with NC-siRNA and exposed to 1,000 PFUs of MHV-3 for 4 h (Yu et al. 2018). Group 3 was exposed to PQQ (0.1 μM) for 6 h, transfected with NC-siRNA, and exposed to 1,000 PFUs of MHV-3 for 4 h. Group 4 was exposed to PQQ (0.1 μM) for 6 h, transfected with Nrf2-siRNA, and exposed to 1,000 PFUs of MHV-3 for 4 h. All transfection experiments were performed using Lipofectamine 2000 (#11668027; Thermo Fisher Scientific) according to the manufacturer’s instructions.

### Western blotting

Total proteins were isolated from liver tissue samples, and immunoblotting was performed as described previously (Xu et al. 2006) using primary antibodies against TNF-α (1:2000) (17590-1-AP; Proteintech Group), FGL2 (1:2000) (#PA5-36390; Thermo Fisher Scientific), Keap1 (1:2000) (#10503-2-AP; Proteintech Group), Nrf2 (1:2000) (ab62352; Abcam), NQO-1 (1:2000) (ab34173; Abcam), heme oxygenase (HO)-1 (1:2000) (#43966; Cell Signaling Technology), and β-actin (1:2000) (#66009-1-Ig; Proteintech Group). All experiments were performed at least three times.

### RNA extraction and reverse transcription-quantitative PCR (RT-qPCR)

Total RNA was extracted from the liver and LESCs using TRIzol reagent (Invitrogen, U.S.A.) and reverse-transcribed using a PrimeScript RT Reagent Kit (Perfect Real Time, Takara Bio Inc.). RT-qPCR was performed as described previously (Jin et al. 2011). All the primers used are listed in Table 1.

**Table 1:** Primers used in this study for real time RT-PCR

Name	S/AS	Sequence
*Keap1*(mouse)	S	CGGGGACGCAGTGATGTATG
	AS	TGTGTAGCTGAAGGTTCGGTTA
*Nrf2* (mouse)	S	TCTTGGAGTAAGTCGAGAAGTGT
	AS	GTTGAAACTGAGCGAAAAAGGC
*NQO-1* (mouse)	S	AGGATGGGAGGTACTCGAATC
	AS	AGGCGTCCTTCCTTATATGCTA
*IL-1β* (mouse)	S	GCAACTGTTCCTGAACTCAACT
	AS	ATCTTTTGGGGTCCGTCAACT
*HO-1* (mouse)	S	AAGCCGAGAATGCTGAGTTCA
	AS	GCCGTGTAGATATGGTACAAGGA
*FGL2*(mouse)	S	GGTGCTCAAAGAAGTGCGGA
	AS	GTTCCTGGACTCTACTGTCCTC
*IL-6* (mouse)	S	CCAAGAGGTGAGTGCTTCCC
	AS	CTGTTGTTCAGACTCTCTCCCT
*GAPDH* (mouse)	S	TGGATTTGGACGCATTGGTC
	AS	TTTGCACTGGTACGTGTTGAT
*Sirt1*(mouse)	S	TCTTGGAGTAAGTCGAGAAGTGT
	AS	GTTGAAACTGAGCGAAAAAGGC

### Microarray analysis

We found the sequence information of hepatitis B virus (HBV)-associated acute liver failure (ALF) and healthy control liver samples on the website https://www.ncbi.nlm.nih.gov/geo/; the reference series was GSE38941. We then analyzed gene expression levels of the Keap1/Nrf2 signaling pathway and generated a heat map.

### Statistical analysis

All experiments and data analyses were carried out at least three times individually in a blinded fashion. Data values are shown as the mean ± standard error of the mean (SEM). Differences between two groups were analyzed using Student’s t-test or one-way ANOVA. Statistical significance was set at *P* < 0.05.

## Results

### Supplementation with PQQ ameliorates MHV-3-induced liver injury.

**Fig. 1:**
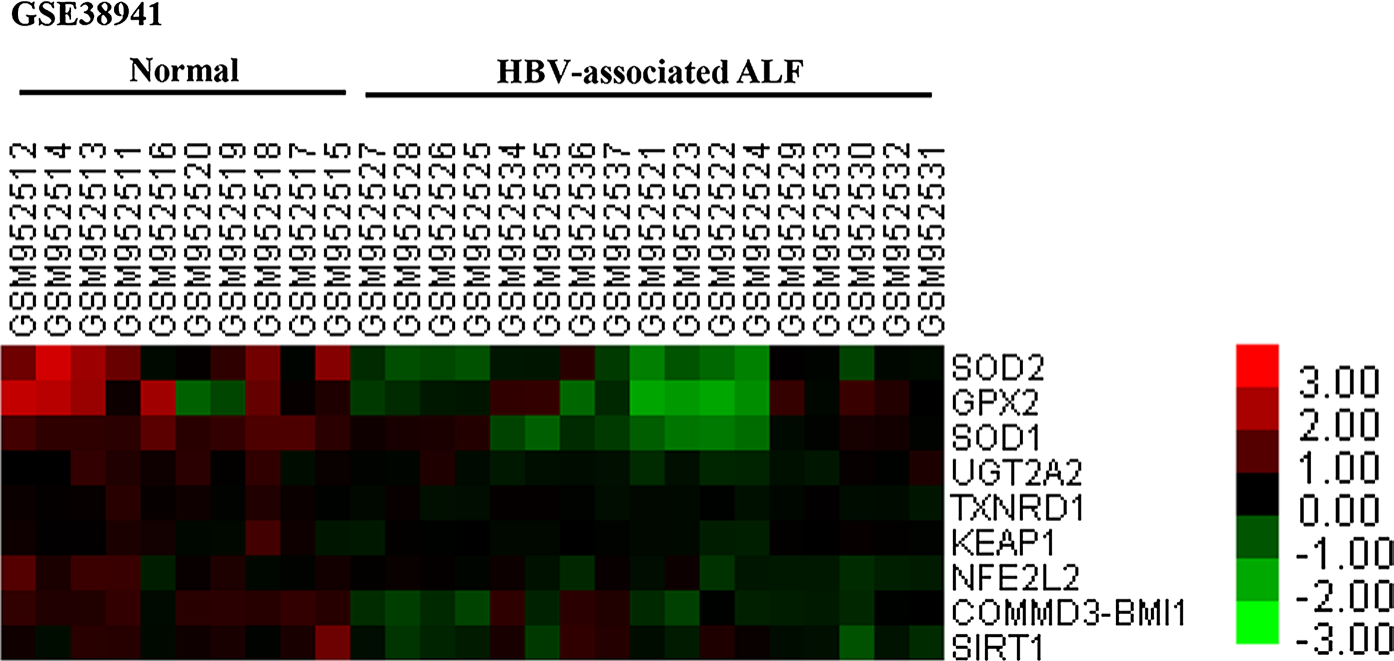
Gene expression profiles of the Keap1/Nrf2 signaling pathway in normal and HBV-associated ALF liver tissue samples. Gene expression profiles were obtained from the website https://www.ncbi.nlm.nih.gov/geo/, and the reference series is GSE38941. There are 10 normal human liver tissue samples and 17 HBV-associated ALF liver tissue samples. Each row represents a single gene, and each column represents a single sample. ALF, acute liver failure; HBV, hepatitis B virus

We first analyzed liver gene expression profiles from normal humans and HBV-associated ALF available at https://www.ncbi.nlm.nih.gov/geo/. The results revealed that both the Keap1/Nrf2 signaling pathway and downstream genes were significantly downregulated in HBV-associated ALF liver tissue samples when compared with that of healthy controls (Fig. 1).

**Fig. 2:**
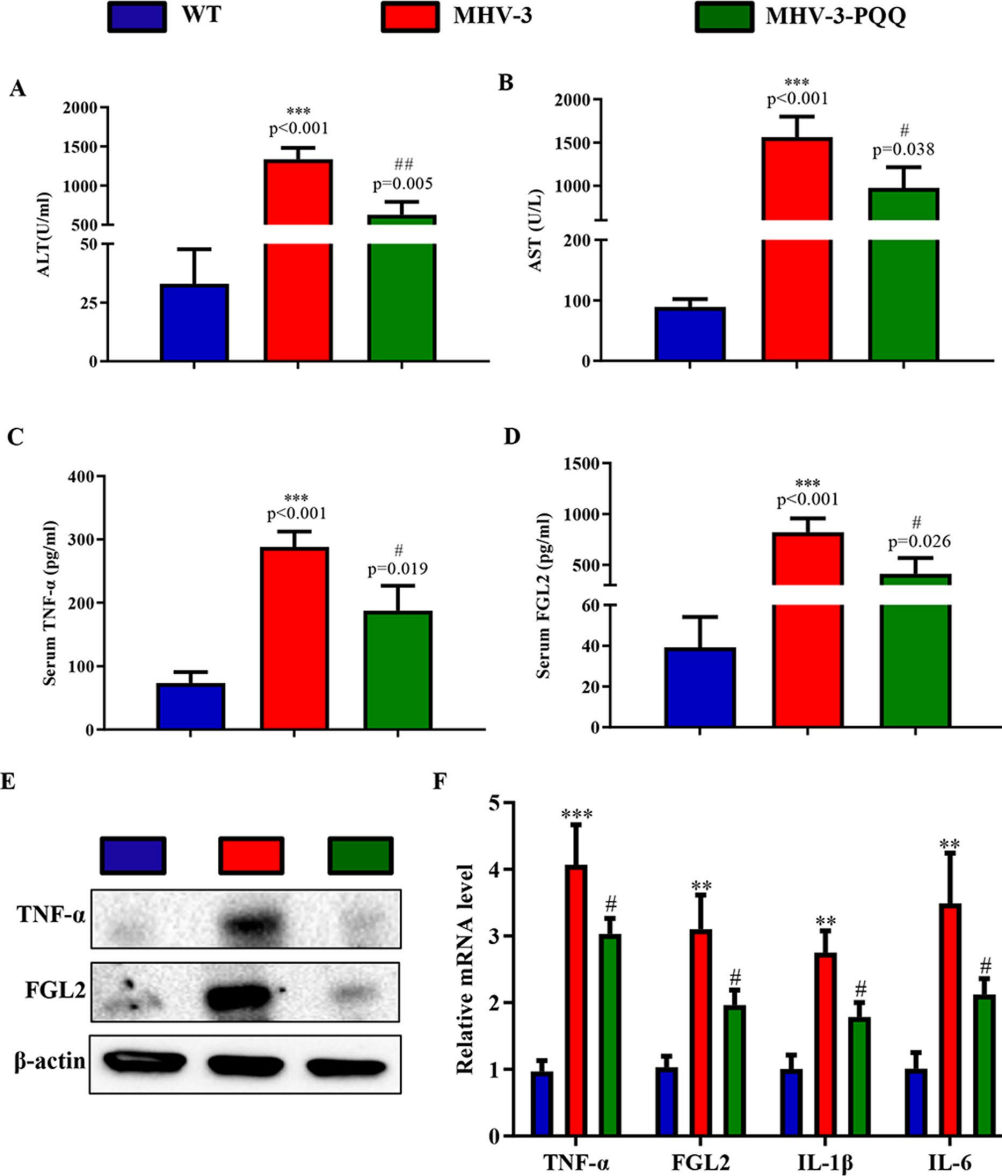
PQQ supplementation attenuates MHV-3-induced hepatitis. Serum levels of **A** ALT, **B** AST, **C** TNF-α, and **D** FGL2 were determined. **E** Western blots of liver tissue showing TNF-α and FGL2 expression. **F** mRNA expression levels of TNF-α, FGL2, IL-1β, and IL-6 in liver tissues were measured by RT-qPCR. Data are presented as mean ± standard error of the mean (SEM), n = 6 mice/group. ***P < 0.001, **P < 0.01 compared with control; #*P* < 0.05, ##*P* < 0.01 compared with MHV-3-induced hepatitis group. *ALT* alanine aminotransferase, *AST* aspartate aminotransferase, *FGL2* fibrinogen-like protein 2, *IL* interleukin, *MHV-3* murine hepatitis virus strain 3, *PQQ* pyrroloquinoline quinone, *TNF-α* tumor necrosis factor-α

We next determined whether PQQ supplementation can decrease MHV-3-induced liver injury. Accordingly, mice groups were fed a normal and PQQ-supplemented diet (4 mg PQQ/kg in normal diet) for 4 weeks before MHV-3 exposure, respectively. Following MHV-3 exposure, PQQ-supplemented mice exhibited milder liver injury than those that received a normal diet, characterized by lower levels of ALT (Fig. 2A), AST (Fig. 2B), TNF-α (Fig. 2C), and FGL2 (Fig. 2D). Following exposure to MHV-3, protein expression levels of TNF-α and FGL2 (Fig. 2E) and RNA expression levels of TNF-α, FGL2, IL-1β, and IL-6 (Fig. 2F) were significantly reduced in PQQ-supplemented mice when compared with those that received a normal diet, as determined by western blotting and RT-qPCR results, respectively. These results suggested that PQQ supplementation can alleviate liver damage induced by MHV-3 exposure.

### PQQ supplementation ameliorates MHV-3-induced liver injury by reducing oxidative stress.

**Fig. 3:**
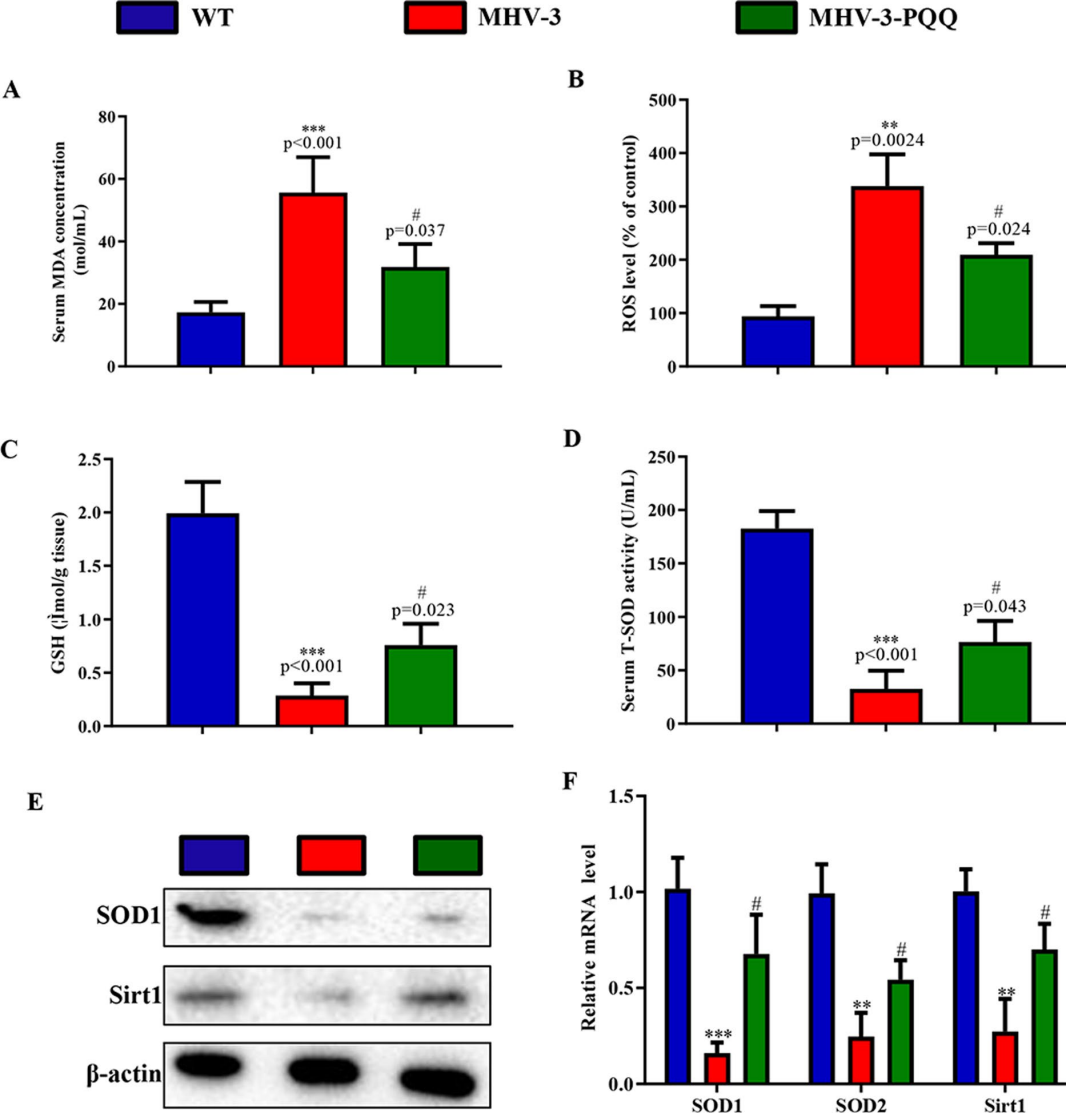
PQQ supplementation attenuates the oxidative stress level in MHV-3-induced hepatitis. Serum levels of **A** MDA and **B** ROS levels in liver tissue were determined. The **C** GSH levels in liver tissue and serum levels of **D** T-SOD were determined. **E** Western blots of liver tissue showing SOD1 and Sirt1. **F** mRNA expression levels of SOD1, SOD2, and Sirt1 in liver tissues were measured by RT-qPCR. Data are presented as mean ± standard error of the mean (SEM), n = 6 mice/group. ***P < 0.001, **P < 0.01 compared to control; #*P* < 0.05, ##*P* < 0.01 compared with MHV-3-induced hepatitis group. *GSH* glutathione, *MDA* malonaldehyde, *MHV-3* murine hepatitis virus strain 3, *PQQ* pyrroloquinoline quinone, *SOD* superoxide dismutase, *T-SOD* total SOD

Oxidative stress is an imbalance that can accelerate the degradation of proteins, lipids, and DNA, and ROS play an important role in the liver fibrogenic response and tissue regeneration (Cichoz-Lach and Michalak 2014). Therefore, to determine whether PQQ ameliorates MHV-3-induced liver injury by reducing oxidative stress, we examined oxidative stress levels in wild-type (WT) control mice, MHV-3-exposed mice, and PQQ-supplemented MHV-3-exposed mice. Following MHV-3 exposure, PPQ-supplemented mice exhibited significantly reduced serum MDA (Fig. 3A) and liver tissue ROS levels (Fig. 3B), as well as significantly increased liver tissue GSH (Fig. 3C) and serum T-SOD levels (Fig. 3D) when compared with mice that received a normal diet. In addition, compared with mice receiving a normal diet and subjected to MHV-3 exposure, PQQ-supplemented MHV-3-exposed mice exhibited significantly increased protein SOD1 and Sirt1 levels (Fig. 3E) and mRNA expression of SOD1, SOD2, and Sirt1 (Fig. 3F) in liver tissues, as determined by western blotting and RT-qPCR results. Therefore, PQQ ameliorated MHV-3-induced liver injury by reducing oxidative stress.

### PQQ supplementation ameliorates the MHV-3-induced hepatic damage by downregulating the Keap1/Nrf2 signaling pathway.

**Fig. 4:**
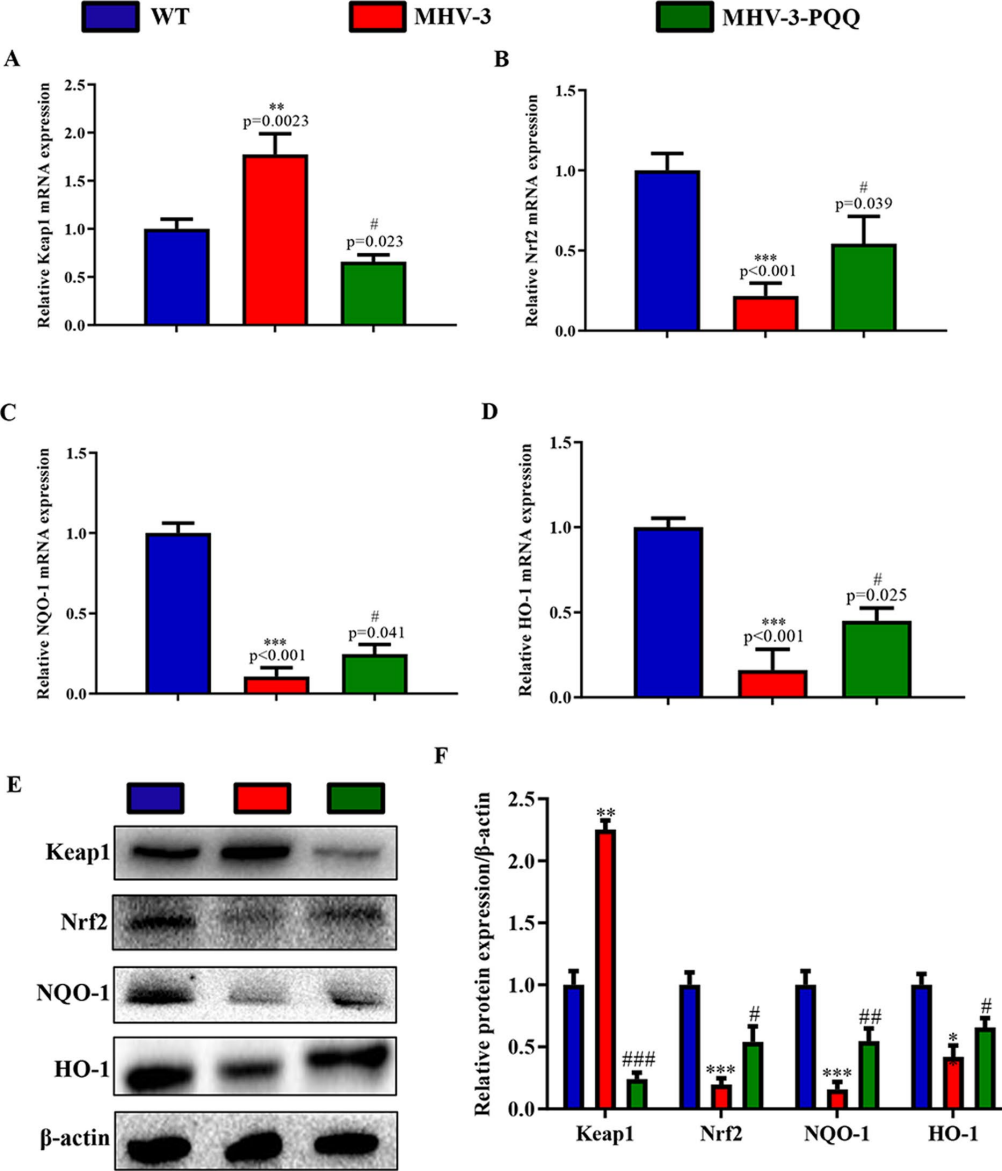
PQQ supplementation attenuates MHV-3-induced hepatitis by inhibiting the Keap1/Nrf2 signaling pathway. mRNA expression levels of **A** Keap1, **B** Nrf2, **C** NQO-1, and **D** HO-1 in liver tissues were measured by RT-qPCR. **E** Western blots of liver tissue showing Keap1, Nrf2, NQO-1 and HO-1. **F** Protein relative levels were analyzed by densitometric analysis. Data are presented as mean ± standard error of the mean (SEM), n = 6 mice/group. ***P < 0.001, **P < 0.01 compared to control; #*P* < 0.05, ##*P* < 0.01, ###*P* < 0.001compared to MHV-3-induced hepatitis group. *HO-1* heme oxygenase-1, *MHV-3* murine hepatitis virus strain 3, *Nrf2* nuclear factor-E2-related factor 2, *PQQ* pyrroloquinoline quinone

To investigate whether the PQQ-mediated improvement in MHV-3-induced liver injury was mediated via the Keap1/Nrf2 signaling pathway, we examined expression levels of Keap1/Nrf2 signaling pathway-associated molecules in WT control mice, MHV-3-exposed mice, and PQQ-supplemented MHV-3-exposed mice. Following MHV-3 exposure, mRNA and protein expression levels of Keap1 were significantly reduced in PQQ-supplemented mice when compared with mice that received a normal diet (Fig. 4A and E). In addition, both mRNA and protein expression levels of Nrf2, NQO-1, and HO-1 in the liver tissue were significantly higher in PQQ-supplemented mice than in those that received a normal diet when exposed to MHV-3 (Fig. 4B–F).

**Fig. 5:**
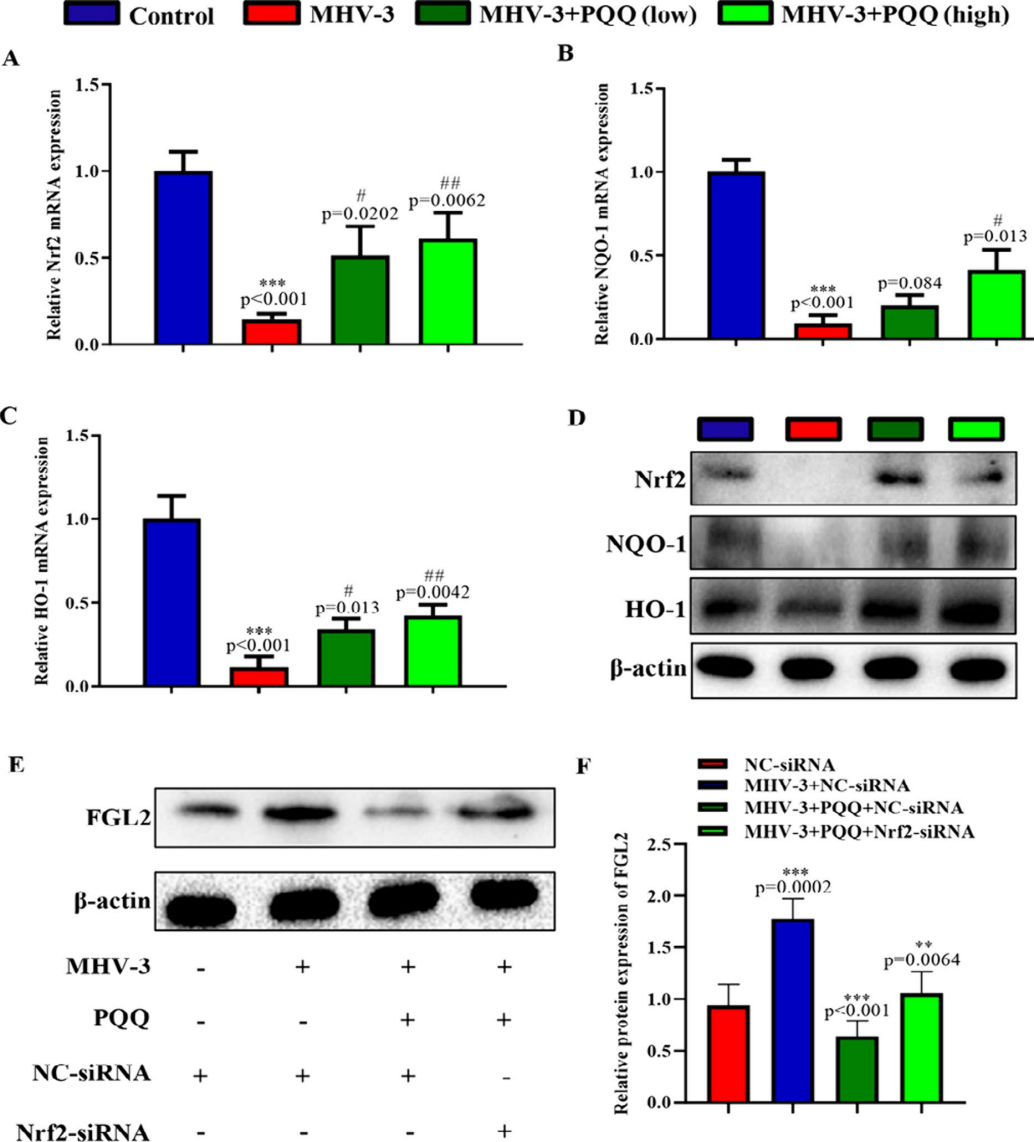
PQQ attenuates over-expression of FGL2 induced by MHV-3 via the Keap1/Nrf2 signaling pathway in LSECs. mRNA expression levels of **A** Nrf2, **B** NQO-1, and **C** HO-1 in LSECs were measured by RT-qPCR when exposed to MHV-3 with or without PQQ. **D** Western blots of Nrf2, NQO-1, and HO-1 in LSECs when exposed to MHV-3 with or without PQQ. **E** Western blots of FGL2 in LSECs when exposed to MHV-3, PQQ or Nrf2-siRNA. **F** FGL2 protein relative levels were analyzed by densitometric analysis. Data are presented as mean ± standard error of the mean (SEM), n = 6 mice/group. ***P < 0.001, **P < 0.01 compared with control; #*P* < 0.05, ##*P* < 0.01 compared with MHV-3-induced hepatitis group. *HO-1* heme oxygenase-1, *FGL2* fibrinogen-like protein 2, *LSECs* liver sinusoidal endothelial cells, *MHV-3* murine hepatitis virus strain 3, *Nrf2* nuclear factor-E2-related factor 2, *PQQ* pyrroloquinoline quinone

To explore this signaling pathway in LSECs, expression levels of Nrf2, NQO-1, and HO-1 were assessed in LSECs isolated from WT mice with or without exposure to MHV-3 or PQQ. Similar to the in vivo results, both mRNA and protein expression levels of Nrf2, NQO-1, and HO-1 in LSECs were significantly reduced in the MHV-3 treatment group when compared with those in the control group; these levels were significantly increased when exposed to PQQ after MHV-3 treatment (Fig. 5A–D). Furthermore, transfection of Nrf2-siRNA in LSECs suppressed the protective role of PQQ (Fig. 5E and F). Therefore, supplementation with PQQ ameliorates MHV-3-induced liver injury by downregulating the Keap1/Nrf2 signaling pathway.

## Discussion

Although studies on the diagnosis and treatment of acute hepatic injury in the past decade have improved survival rates, the overall rates of progression and prognosis of acute liver injury remain unclear. Elucidating molecular mechanisms related to the pathophysiology of acute liver injury will facilitate early-stage diagnosis and allow the development of additional therapeutic targets. PQQ is a new type of anionic water-soluble redox compound with neuroprotective and free-radical-scavenging properties and anti-inflammatory activity (Huang et al. 2017; Zhang et al. 2020a, b; Ouchi et al. 2013). PQQ (20 mg per day) was administered for 12 weeks to the adults aged between 20 and 65 years, the participants showed improvements in composite memory and verbal memory (Tamakoshi et al. 2023). To date, there have been no reports on the use of PQQ for MHV-3-induced FH. Therefore, in the present study, we aimed to investigate the protective effect of PQQ against MHV-3-induced FH via the Keap1/Nrf2 signaling pathway-mediated antioxidant stress response.

Currently, drug treatments for liver injury are limited. N-acetylcysteine can be employed to treat acetaminophen-induced liver injury to promote recovery and reduce the need for liver transplantation. Therefore, the search for new and effective medications with fewer side effects is important. When oxygen free radicals in the body are unbalanced, or the excess oxidant is taken up, ROS or related substances accumulate excessively in the body, potentially causing various cellular toxic effects, resulting in reversible or irreversible damage (Fay et al. 1999). Under normal physiological conditions, various antioxidants and antioxidant enzymes can scavenge excess free radicals and prevent damage to the body. Free radical-induced biofilm damage can cause intracellular environmental disorders, cell aging, and cell death. Lipid peroxidation can inactivate ribonucleic acid, lead to DNA and RNA linkage, and trigger DNA mutations (Coia et al. 2018). In addition, lipid peroxidation generates aldehydes, which can combine with phosphoric acid and proteins to form lipofuscin, and brain deposition leads to mental retardation (Altinoz and Ozpinar 2019). Therefore, oxidative stress damage in the liver of patients with acute liver damage plays an important role in apoptosis, necrosis, and the pathogenesis of acute liver damage.

Nrf2 acts as an important regulator of cellular redox stress. Upon stimulation, Nrf2 shifts from the cytoplasm to the nucleus and binds to antioxidant response elements (AREs) to promote the expression of downstream proteins, including NQO-1, HO-1, and glutathione peroxidase 3 (GPx-3), and reduces sensitivity to oxidative stress injury (Chen et al. 2006). This function is considered an important therapeutic target for oxidative stress-related diseases. Shi et al. have reported that chlorogenic acid affords protection against acute liver injury by activating the Nrf2 pathway (Shi et al. 2018), and Zhao et al. have found that polydatin exerts anti-inflammatory effects via the Keap1/Nrf2 pathway (Zhao et al. 2018). Hence, the Keap1/Nrf2 signaling pathway can be considered a potential therapeutic target to prevent MHV-3-induced FH in the present study. Our results revealed that PQQ treatment relieved liver damage induced by MHV-3. PQQ led to a decrease in the expression level of Keap1 and an increase in Nrf2, HO-1, and NQO1 levels in the hepatic samples of MHV-3-treated mice. Furthermore, knockdown of Nrf2 expression in LESCs abrogated the protective effects of PQQ against MHV-3-induced liver damage.

In conclusion, our findings demonstrated that PQQ treatment could alleviate inflammatory and oxidative stress damage in liver tissue cells by repressing the Keap1/Nrf2 signaling pathway, ultimately mitigating MHV-3-induced acute liver injury. Therefore, PQQ could be a potential therapeutic agent for treating ALF.

## Data Availability

The raw data supporting the conclusions of this article will be made available by the authors, without undue reservation.

## Declarations

### Research involving human and animal participants

All experimental procedures followed the ARRIVE guidelines, were approved by the Committee on the Ethics of Animal Experiments (IACUC2001020), and were in accordance with the guidelines of laboratory mouse research at Nanjing Medical University.
